# Effect of Fatigue on Hamstring Reflex Responses and Posterior-Anterior Tibial Translation in Men and Women

**DOI:** 10.1371/journal.pone.0056988

**Published:** 2013-02-27

**Authors:** Martin Behrens, Anett Mau-Moeller, Franziska Wassermann, Sven Bruhn

**Affiliations:** Department of Exercise Science, University of Rostock, Rostock, Germany; The University of Tennessee Health Science Center, United States of America

## Abstract

Anterior cruciate ligament (ACL) rupture ranks among the most common injuries in sports. The incidence of ACL injuries is considerably higher in females than in males and the underlying mechanisms are still under debate. Furthermore, it has been suggested that muscle fatigue can be a risk factor for ACL injuries.

We investigated gender differences in hamstring reflex responses and posterior-anterior tibial translation (TT) before and after fatiguing exercise. We assessed the isolated movement of the tibia relative to the femur in the sagittal plane as a consequence of mechanically induced TT in standing subjects. The muscle activity of the hamstrings was evaluated. Furthermore, isometric maximum voluntary torque (iMVT) and rate of torque development (RTD) of the hamstrings (H) and quadriceps (Q) were measured and the MVT H/Q as well as the RTD H/Q ratios were calculated.

After fatigue, reflex onset latencies were enhanced in women. A reduction of reflex responses associated with an increased TT was observed in females. Men showed no differences in these parameters. Correlation analysis revealed no significant associations between parameters for TT and MVT H/Q as well as RTD H/Q.

The results of the present study revealed that the fatigue protocol used in this study altered the latency and magnitude of reflex responses of the hamstrings as well as TT in women. These changes were not found in men. Based on our results, it is conceivable that the fatigue-induced decrease in neuromuscular function with a corresponding increase in TT probably contributes to the higher incidence of ACL injuries in women.

## Introduction

Anterior cruciate ligament (ACL) rupture ranks among the most common injuries in sports [1] and is associated with long recovery times and high socio-economic costs. The incidence of ACL injuries is considerably higher in females than in males and the underlying mechanisms are still under debate [2]. It has been argued that differences in the passive and active stability of the tibiofemoral joint could be responsible for the higher injury rate. The passive stability of the knee joint depends largely on the laxity of the ligaments and the geometry of the articular surfaces. Active stability relies on the patellar tendon-tibia shaft angle, muscle activity pattern, muscle reaction time, time to peak torque and muscle stiffness [3].

Furthermore, it has been suggested that muscle fatigue can be a risk factor for ACL injuries [2], [3]. Several studies have shown that muscle fatigue is associated with decreased joint proprioception and postural stability as well as increased joint laxity [4]–[6]. Fatigue has also been shown to alter the control of lower extremity mechanics during landing, side-step cutting and running [7]–[10]. Epidemiological data suggest that injury rates tend to be higher at the end of matches [11], [12], suggesting fatigue could be related to injury. Therefore, fatigue may play an important role in the pathomechanics of knee joint injuries [13].

Several studies have focused on hamstring reflex responses and their role in resisting posterior-anterior tibial translation (TT), which served as a criterion for functional knee stability [14], [15]. A study by Friemert et al. [14] has revealed that these reflex responses originate from primary and secondary spindle afferents in the hamstring muscles. Some studies have investigated the effect of fatigue on reflex responses of the hamstring muscles and TT [13], [16]. These studies have revealed that fatigue can modulate the timing and magnitude of reflex activity as well as increase TT. However, the effect of fatigue on gender-specific hamstring reflex responses and TT has not been sufficiently investigated.

Furthermore, it has been suggested that a low hamstrings/quadriceps (H/Q) strength ratio can be an indicator for knee joint injury risk [17]–[19]. According to a study by Krosshaug et al. [20], ACL injuries occur between 17 and 50 ms after initial ground contact. Therefore, Zebis et al. [19] introduced a H/Q ratio that takes the ability of the subject to rapidly develop force within 50 ms into account. The authors proposed that this rate of torque development (RTD) H/Q ratio could be used in addition to the traditional H/Q ratio, derived from the peak force values during maximum voluntary contraction (MVC), to describe the potential for knee joint stabilization.

The purpose of the present study was to analyze gender differences in hamstring reflex responses and TT before and after a fatigue protocol. We assessed the isolated movement of the tibia relative to the femur in the sagittal plane as a consequence of mechanically induced TT in standing subjects. The muscle activity of the lateral and medial hamstrings was evaluated. Furthermore, isometric maximum voluntary torque (iMVT) and RTD of the hamstrings and quadriceps were measured and MVT H/Q as well as RTD H/Q ratios were calculated.

It was hypothesized that, due to fatigue, reflex components of the muscles are impaired and TT is altered. In addition, we presumed that there is an association between TT and the MVT H/Q ratio as well as the RTD H/Q ratio. We assumed that the main outcome variables would show gender-specific differences.

## Methods

### Subjects and study design

**Figure 1:**
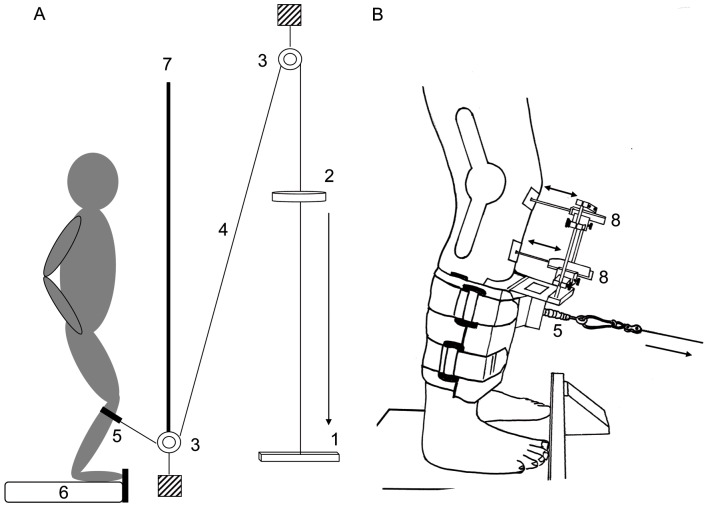
Schematic drawing of the experimental setup. A: Experimental setup, B: Measurement system **|** 1: stopper, 2: falling weight, 3: pulley, 4: steel rope, 5: force transducer, 6: force plate, 7: visual cover, 8: linear potentiometer. Arrows indicate the direction of the force. Posterior-anterior tibial translation was assessed by two linear potentiometers (8) placed on the patella and the tibial tuberosity. A force transducer (5) was used to measure the force transmitted to the shank.

Fifty healthy subjects (25 males: 25.4±2.7 years, 78.8±7.8 kg, 180.7±5.3 cm/25 females: 23.3±2.2 years, 64.9±8.9 kg, 168.3±3.6 cm) with no history of neurological disorders or injuries participated. Before testing, subjects were instructed to refrain from consuming alcohol and caffeine in the 24 h preceding the experiment and not to perform any strenuous exercise in the 48 h prior to the measurements. All persons signed informed consent. The study was conducted according to the declaration of Helsinki and was approved by the ethics committee of the University of Rostock (A 2011 129). During the experiment, participants were examined with regard to reflex responses and TT before and after a fatiguing jumping task. The measurements were performed using a knee arthrometer [16], [21]–[23] (Fig. 1). In addition, before the execution of the fatigue protocol, the subjects performed isometric MVCs for the hamstrings and the quadriceps using a dynamometer. The experiment required approximately 2 h per person.

### Measurement of posterior-anterior tibial translation

Participants were examined in bipedal stance with the knees in 30° flexion (0° = full extension). In order to standardize the stance position between the trials, subjects stood on a force plate (sampling frequency: 40 Hz, GKS 1000^®^, IMM Holding GmbH, Germany). Subjects were thereby provided with online feedback about their center of pressure. Furthermore, to avoid the influence of acoustic signals on the subjects they wore ear protection (Bilsom Thunder T3). The subjects had no information on the point in time of the perturbation. A standardized force was applied to the proximal shank of the dominant leg using a pulley system in order to induce TT. A device was attached to the tibia to secure two linear potentiometers (measuring accuracy: <0.01 mm, linearity: ±0.7%, Type CLR13–50; Megatron, Germany) that were placed on the patella and the tibial tuberosity (Fig. 1). The knee arthrometer enabled us to measure the translational movement of the tibia relative to the femur in the sagittal plane. The interface pressure between the knee arthrometer and the subjects' tibia was controlled by an air pressure recorder (Kikuhime, TT MediTrade, Denmark). The interface pressure was kept constant before and after the fatiguing exercise. The locations of the subjects' feet on the force plate, the stabilizing device and the linear potentiometers were marked in order to ensure the same positions before and after the fatigue protocol. The applied force that induced TT was controlled using a force transducer (measuring range: 0–5000 N, sensitivity: −3.42 to 3.36 pC·N^−1^, linearity: ±0.2–0.3%; Kistler, Switzerland). The force sensor was placed between the stabilizing device and the pulley system. TT was elicited 15 times in order to familiarize the subject with the measurement. Thereafter, further 15 perturbations were applied before and immediately after the fatigue protocol. The inter stimulus interval was varied between ∼8 s and ∼14 s to avoid anticipation.

### EMG and torque recordings

Surface EMG was recorded using bipolar Ambu® Blue Sensor N electrodes. The electrodes were attached to the shaved, abraded and cleaned skin over the biceps femoris (BF) and semitendinosus/semimembranosus (ST) of the dominant leg (resistance between electrodes <5 kΩ). The electrodes were applied with a center-to-center distance of 2 cm over the muscle bellies and in line with the presumed direction of the underlying muscle fibers. The reference electrode was attached to the patella. Signals were amplified (2500x), band-pass filtered (10–1300 Hz) and digitized (sampling frequency: 5 kHz) through an analog-to-digital converter (DAQ Card™-6024E, National Instruments, USA).

Torque was measured using a CYBEX NORM dynamometer (Computer Sports Medicine®, Inc., Stoughton, MA). A hip joint angle and knee joint angle of 30°, respectively (0° = full extension), was chosen in order to mimic the posture and therefore the length of the muscles during the measurement of TT. The axis of the dynamometer was aligned with the anatomical knee flexion-extension axis and the lever arm was attached to the anterior aspect of the shank 2–3 cm above the lateral malleolus. Straps across the waist and chest prevented excessive movements. The iMVT was tested by asking the subjects to exert isometric knee extensions and flexions against the lever arm of the dynamometer for 3 s. For each trial, subjects were thoroughly instructed to act as forcefully and as fast as possible. They were motivated by strong verbal encouragement and online visual feedback of the instantaneous dynamometer torque provided on a digital oscilloscope (HM1508, HAMEG Instruments, Germany). Care was taken that the iMVT trials were performed without an apparent countermovement or pre-tension (change in baseline torque <0.5 Nm during 200 ms prior to contraction onset). A rest period of 2 min was allowed between trials. The maximal attempts were recorded until the coefficient of variance of five consecutive trials was below 5% [24]. The EMG, linear potentiometer, force and torque signals were stored on a hard drive for later analysis with custom built LABVIEW^®^ based software (Imago, Pfisoft, Germany).

### Fatigue protocol

Fatigue was induced by repetitive jumping performed between ∼90° and 0° knee flexion (0° = full extension). The fatigue protocol consisted of consecutive maximal countermovement jumps [25], each one separated by 4 s according to the sound of a digital metronome. The jumps were performed until the subjects reached a fatigued state defined as the inability to reach 50% of their maximal jump height for 3 consecutive jumps or until the subjects reached an intolerable state of dyspnea or exhaustion. Rate of perceived exertion (RPE) was assessed using the Borg 6–20 scale.

### Data analysis

**Figure 2:**
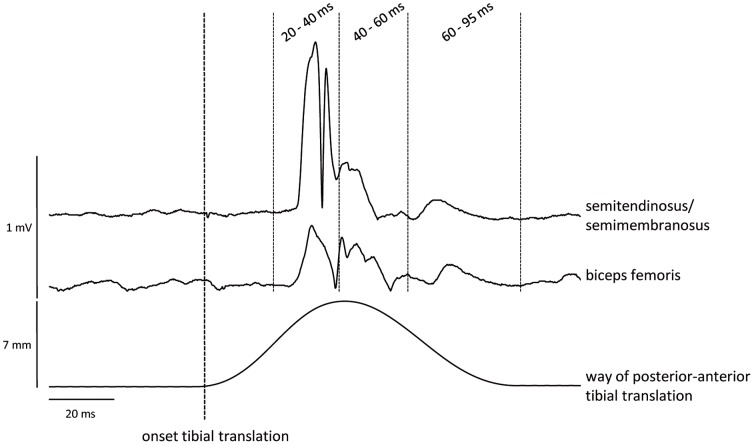
EMG and tibial translation data from one representative subject. EMG activity of biceps femoris and semitendinosus/semimembranosus as well as posterior-anterior tibial translation of one subject. In the figure, EMG data is rectified in order to visualize the different parts of the hamstring stretch reflex. The vertical bold line indicates the onset of posterior-anterior tibial translation. Three different time intervals were analyzed (20–40, 40–60 and 60–95 ms).

In order to analyze the data, the EMG signals of each subject were averaged. The EMG onset latencies were defined as the time between onset of TT and onset of significant muscular activity, e.g. the beginning of EMG deflection (average EMG baseline value measured over 100 ms ± 3 standard deviations). Muscle activity was analyzed according to Bruhn et al. [23], that is, the TT signal indicated the onset of perturbation and muscle activity was calculated over different time intervals relative to the onset of TT, i.e. 20–40, 40–60 and 60–95 ms (Fig. 2), using the root mean square of the EMG signal (RMS-EMG). In order to assess background activity before and after fatigue, RMS-EMG was calculated over 50 ms prior to the onset of TT. Consequently, background activity was subtracted from the reflex responses. Maximum TT was determined based on the TT curves.

The torque signals were corrected for the effect of gravity and the three best maximum voluntary contractions were retained for analysis. The iMVT was defined as the highest peak torque value. Explosive voluntary muscle strength was determined by analyzing the average RTD over time intervals of 0–50, 0–100 and 0–200 ms relative to the onset of contraction. The identification of torque onset was made manually according to the method of Tillin et al. [26]. It has been suggested that this is the best method for detecting signal onsets [27]. MVT H/Q as well as RTD H/Q (RTD_0–50_, _0–100, 0–200_ H/Q) ratios were calculated as follows [19]: MVT H/Q = Hamstrings MVT/Quadriceps MVT and RTD_x_ H/Q = Hamstrings RTD_x_/Quadriceps RTD_x_, where x denotes the analyzed time interval (0–50, 0–100, 0–200 ms).

### Statistical analysis

Data were checked for normal distribution using the Kolmogorov-Smirnov test. Differences between the values before and after the fatigue protocol were tested for significance by repeated measures ANOVA. Differences between the groups were tested for significance by the unpaired Student's t test. Correlations between parameters were calculated using Pearson's correlation coefficient. In each case the level of significance was established at p≤0.05. SPSS 20.0 (SPSS Inc., Chicago, IL, USA) was used for statistical analysis. Effect size (ES) was calculated with the statistical software package G*Power (Version 3.1.5) [28]. The ES characterizes the effectiveness of an intervention. Furthermore, it is used to determine whether a statistically significant difference is a difference of practical importance. ES-values = 0.10 indicate small, ES = 0.25 medium and ES = 0.40 large effects [29]. Data are presented as group mean values ± standard error of the mean in the figures and as group mean values ± standard deviation in the table.

## Results

**Figure 3:**
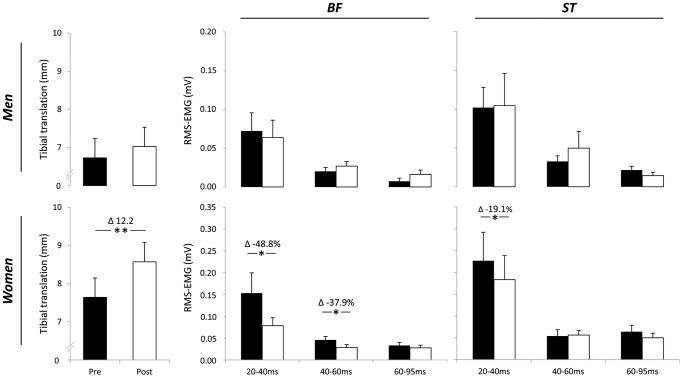
Effect of fatigue on tibial translation (left) and reflex responses (middle and right). Filled bars: Pre, open bars: Post, BF: biceps femoris, ST: semitendinosus/semimembranosus. Data are displayed as means ± standard error of the mean. * denotes a significant difference compared to the pre-measurement, * *P*≤0.05; ** *P*≤0.01.

During the fatiguing exercise, men performed 159.8±90.9 and women 150.6±62.4 jumps. RPE using the Borg 6–20 scale was 15.2±2.0 for males and 14.8±1.6 for females. The force applied to the proximal shank of the dominant leg remained constant during the pre- and post-test (Table 1). Reflex onset latencies of BF and ST were significantly delayed in women at post-test (Table 1). BF muscle activity decreased after fatigue in women in the time intervals 20–40 ms (*F* = 4.99, *P* = 0.035, η^2^ = 0.166, ES = 0.45) and 40–60 ms (*F* = 7.22, *P* = 0.013, η^2^ = 0.224, ES = 0.54). The reflex response of BF between 60–95 ms (*F* = 0.455, *P* = 0.506, η^2^ = 0.018, ES = 0.14) was unchanged in women after the fatigue protocol. The reflex activity of ST was significantly reduced after fatigue between 20–40 ms in women (*F* = 5.38, *P* = 0.029, η^2^ = 0.183, ES = 0.47), but not between 40–60 ms (*F* = 0.051, *P* = 0.823, η^2^ = 0.002, ES = 0.045) and 60–95 ms (*F* = 3.001, *P* = 0.096, η^2^ = 0.111, ES = 0.35). TT increased significantly after the fatigue protocol in females (*F* = 11.86, *P* = 0.002, η^2^ = 0.322, ES = 0.69) (Fig. 3).

**Table 1:** Force applied to the proximal shank of the dominant leg and EMG onset latencies before and after the fatigue protocol for men and women. In addition, weight-normalized isometric maximum voluntary torque (iMVT), weight-normalized rate of torque development (RTD_0–50, 0–100, 0–200_) of the hamstrings (H) and quadriceps (Q), MVT H/Q ratio and RTD H/Q ratios (RTD_0–50, 0–100, 0–200_ H/Q) for men and women.

Parameter	Men						Women			
	*Pre*		*Post*		*P*		*Pre*	*Post*		*P*
Force (N)	249.73±11.48		243.74±22.32		NS (0.220)		241.48±13.53	240.15±12.29		NS (0.468)
EMG onset latencies (ms)										
*BF*	23.60±2.57		24.59±2.17		NS (0.080) ES = 0.47		22.10±2.23	23.05±2.56 *		0.022 (ES = 0.57)
*ST*	22.92±2.59		24.92±2.27		NS (0.058) ES = 0.61		22.37±2.30	23.42±2.54 *		0.017 (ES = 0.62)
	*H*			*Q*		*H*			*Q*	
iMVT (N·m·kg^−1^)	1.21±0.20 ††			2.07±0.35 ††		0.87±0.16 ††			1.79±0.33 ††	
RTD (N·m·s^−1^·kg^−1^)										
0–50 ms	2.76±2.77			10.80±6.61		1.72±1.01			7.37±4.38	
0–100 ms	5.72±2.06 ††			10.93±4.18 †		3.75±1.42 ††			8.29±3.18 †	
0–200 ms	4.70±0.94 ††			7.90±2.00		3.32±0.77 ††			6.83±1.73	
	*H/Q*					*H/Q*				
MVT H/Q ratio	0.59±0.08 ††					0.50±0.10 ††				
RTD H/Q ratio										
0–50 ms		0.50±0.53				0.46±0.71				
0–100 ms		0.63±0.43				0.51±0.25				
0–200 ms		0.62±0.16 †				0.50±0.12 †				

BF: biceps femoris, ST: semitendinosus/semimembranosus. * denotes a significant difference compared to the pre-measurement, * *P*≤0.05. **†** indicates a significant difference between men and women, **†** *P*≤0.05; **††** *P*≤0.01. ES = effect size. Values are means ± standard deviation.

No statistical differences in the reflex responses of BF between 20–40 ms (*F* = 0.601, *P* = 0.448, η^2^ = 0.032, ES = 0.18), 40–60 ms (*F* = 4.118, *P* = 0.057, η^2^ = 0.186, ES = 0.48) and 60–95 ms (*F* = 1.429, *P* = 0.247, η^2^ = 0.074, ES = 0.28) were observed in men. The reflex activity of ST was not altered in the time intervals 20–40 ms (*F* = 0.008, *P* = 0.930, η^2^ = 0.000, ES = 0.00), 40–60 ms (*F* = 0.806, *P* = 0.382, η^2^ = 0.045, ES = 0.22) and 60–95 ms (*F* = 1.744, *P* = 0.204, η^2^ = 0.093, ES = 0.32). TT did not change following the fatigue protocol in males (*F* = 0.932, *P* = 0.346, η^2^ = 0.047, ES = 0.22).

The weight-normalized iMVT of the hamstring and the quadriceps muscle was significantly higher in men than in women (*P*≤0.001). Furthermore, weight-normalized RTD_0–100_ and RTD_0–200_ of the hamstrings (*P*≤0.01) but only RTD_0–100_ of the quadriceps (*P*≤0.05) were significantly higher in males than in females. The MVT H/Q ratio and the RTD_0–200_ H/Q ratio were significantly different between men and women (*P = *0.003 and *P = *0.013, respectively) (Table 1). Correlation analysis for all participants revealed no significant associations between the parameters for TT and the MVT H/Q ratio as well as RTD H/Q ratio (Table 2).

**Table 2:** Correlations between posterior-anterior tibial translation and strength parameters for all subjects.

Parameter	MVT H/Q ratio	RTD_0–50_ H/Q ratio	RTD_0–100_ H/Q ratio	RTD_0–200_ H/Q ratio
Tibial translation Pre	−0.07	0.10	0.25	0.09
Tibial translation Post	−0.24	−0.09	0.11	−0.03
Tibial translation diff.	−0.28	−0.30	−0.21	−0.19

Tibial translation diff. stands for the difference between the tibial translation before and after fatigue.

## Discussion

The purpose of this study was to elucidate the effect of fatigue, induced by repetitive jumping, on reflex activity of the hamstrings and TT in men and women. In addition, we presumed that there is an association between the extent of TT and the MVT H/Q ratio as well as the RTD H/Q ratio.

Reflex onset latencies were enhanced in women after the fatiguing task. The results revealed a fatigue-induced reduction of reflex responses in women associated with an increased TT. Men showed no significant differences in the parameters after the fatigue protocol. Correlation analysis revealed no significant associations between the parameters for TT and the MVT H/Q ratio as well as the RTD H/Q ratio.

### Tibial translation

It has been assumed that increased joint laxity may contribute to increased ACL injury risk [30]. Several studies have found significant increases in anterior knee laxity, for example, after running [31]–[33] or a regular workout in volleyball [34]. However, only Kvist et al. [34] have compared males and females after fatiguing exercise and found an increase in TT for men. Nevertheless, these studies measured anterior knee laxity while subjects were relaxed. In contrast, in the current study TT was measured in a functional weight-bearing situation. In a situation such as this, axial loading and forces due to muscle contraction could reduce rotation and translation compared to the passive condition [35], [36]. The present study found an increase in TT in women but not in men after the fatiguing exercise. The ES of 0.69 indicates that the fatigue protocol induced a large effect regarding the parameter TT in females. Studies using a similar methodology have shown that an isokinetic fatigue protocol performed with a dynamometer can increase TT [13], [16]. However, only the study by Wojtys et al. [13] has focused on gender-specific differences but it used a very small sample size (six men and four women). The authors reported no gender difference in any parameter.

In general, knee ligamentous structures probably undergo some increase in laxity during exercise, thereby placing athletes at risk for ligamentous injury [6]. It is assumed that this is due to the fact that joint structures, particularly the ligaments, exhibit viscoelastic properties [37]. Therefore, cyclic stress of the ligamentous structures leads to time-dependent and stress-dependent modifications and therefore increased ligamentous laxity [6], [37]. However, the muscles that cross the knee joint play a large role in maintaining physiological kinematics of the knee. Muscle activity is able to induce large changes in strains as well as forces experienced by the ACL [38].

### Reflex responses

The fast activation of muscles by means of reflexes may play a substantial role in the stabilization of the knee joint [14]. It has been suggested that the direct reflex arc between the ACL and the hamstrings makes only a minor contribution to the biphasic reflex response in the hamstring muscles [39]. Therefore, it has been suspected that the reflex response is mainly generated by hamstring stretch reflexes [15]. In the current study, delayed reflex onset latencies for BF and ST were found in women after the fatigue protocol. The ES for the parameters (BF = 0.57 and ST = 0.62) indicate that the fatiguing exercise had a large effect on the latencies of both muscles. The onset latencies for BF and ST in men were not statistically different after fatigue. However, ES of 0.47 and 0.61, respectively, indicate that the fatigue protocol provoked a large effect for men as well. Similar results were reported by Melnyk and Gollhofer [16] who found an increased latency of reflex responses after submaximal fatigue. The authors have argued that the slowing of reflex responses probably does not play a substantial role in functional knee stability. Nevertheless, the authors have not focused on gender-specific differences.

In the current study, the reflex response of the females was significantly reduced in BF for the time intervals 20-40 and 40–60 ms (ES = 0.45 and ES = 0.54, respectively). The same was true for ST from 20–40 ms (ES = 0.47). The results of the present study correspond with the results of Melnyk and Gollhofer [16] who found significantly decreased iEMG values for the short latency response and medium latency response of the hamstring stretch reflex after an isokinetic concentric-eccentric fatigue protocol. As before, gender-specific differences were not investigated. Moore et al. [40] have investigated vastus lateralis reflex activity induced by a standardized tendon tap with a spring-loaded reflex hammer before and after fatiguing isokinetic contractions. The authors reported a significant increase in reflex amplitude in men and a tendency to a reduction in women. They concluded that males and females might respond differently to fatigue. A reason for this could be that men and women activate their muscles differently according to the requirements of the movement task. For example, Rozzi et al. [41] have observed that women show greater muscle activity of the lateral hamstring muscle when landing from a jump, and possess increased knee joint laxity as well as longer time to detect knee joint motion compared with men. The authors concluded that the greater EMG peak amplitude and area in women might be an attempt of the nervous system to compensate for the greater joint laxity and proprioceptive deficit. Furthermore, the authors argued that an interruption of this compensatory mechanism, for example due to fatigue, might increase joint laxity that may cause ligament injury. The greater muscle activity of the hamstrings when landing from a jump in females may be an explanation for the differing results in the present study between men and women regarding reflex responses and TT. It is conceivable that the fatigue protocol used in this study, which consisted of repetitive jumps performed until exhaustion, induced more fatigue in the hamstring muscles of women and therefore impaired hamstring reflex responses.

The decrease in muscle activity in distinct time intervals in women could be explained by different physiological processes: (i) fatigue-induced changes in intrafusal properties, (ii) presynaptic inhibition (PSI) of Ia afferents and (iii) changes in intrinsic properties of motoneurons. Fatigue-induced changes in intrafusal properties are assumed to occur during sustained MVCs and probably reduce intrafusal contraction force and thereby the fusimotor-driven afferent discharge [42]. Furthermore, it has been suggested that submaximal isometric fatiguing exercise is also able to change intrafusal properties and, in turn, reflex responses [43]. Another explanation might be the decline in transmission from Ia afferents to motoneurons due to PSI mediated by group III and IV afferents [44]. These afferents are sensitive to several parameters associated with either metabolic fatigue or muscle damage [45]. It has been found that these afferents have a powerful input to inhibitory interneurons which induce PSI of Ia afferent terminals [46]. In addition, the possibility of changes in intrinsic properties of motoneurons should be taken into account [47]. It has been found that motoneurons can experience an intrinsic adaptation in firing frequency to a constant excitatory drive [48]. The decrease in jump height below 50% during the fatigue protocol and the RPE indicated that the jumping exercise was exhaustive. Therefore, it can be assumed that the exercise caused a stressful metabolic loading and thereby stimulated group III and IV afferents that probably induced PSI of Ia afferents. Furthermore, it is conceivable that the reflex inhibition of the motoneuron pool was accompanied by changes in the intrinsic properties of motoneurons.

The results of the present study revealed that the fatigue protocol used in this study altered the latency as well as magnitude of reflex responses of the hamstring muscles and TT in women. These changes were not found in men. The authors of various studies have suggested that the hamstring muscles play an important role in maintaining knee stability and that they protect the ACL during movements of the tibia relative to the femur [49]–[51]. Therefore, decreased reflex responses of the hamstring muscles and in turn an increased TT might contribute to the pathomechanics of knee joint injuries. It has been shown that female athletes have an increased risk for ACL injuries [2]. Based on our results it is conceivable that the fatigue-induced decrease in neuromuscular function with a corresponding increase in TT probably contributes to the higher incidence of ACL injuries in women.

### Limits of the study

The measurements used in this study were performed in a functional weight-bearing situation. Only a few studies have investigated the effect of fatigue on tibial translation and muscular responses with a similar methodology [13], [16]. Therefore, comparisons with studies that used another methodology, e.g. experiments that measured anterior knee laxity while subjects were relaxed [31]–[33], should be viewed with caution. Furthermore, a study by Bruhn et al. [23] has revealed that hamstring reflex responses are modulated according to the stimulus characteristics, i.e. stretch velocity vs. stimulus amplitude. It is possible that men and women respond differentially with regard to stimulus characteristics. In this respect, it is noteworthy that body weight may have influenced the response to the constant stimulus and in turn the observed differences in the neuromuscular response and TT. Moreover, we assume that the fatigue protocol used in this study caused a stressful metabolic loading and thereby modulated the reflex responses. Unfortunately, we have not measured metabolic data that could support this assumption.
